# A Categorical ANCOVA Approach to Severity Endophenotype-Specific Genome-Wide Association Studies in Childhood Asthma

**DOI:** 10.3390/jpm16010032

**Published:** 2026-01-05

**Authors:** Shraddha Piparia, Parham Hadikhani, John Ziniti, Julian Hecker, Alvin T. Kho, Rinku Sharma, Juan C. Celedón, Michael J. McGeachie, Scott T. Weiss, Kelan G. Tantisira

**Affiliations:** 1 Division of Pediatric Respiratory Medicine, University of California San Diego, San Diego, CA 92123, USA; 2 Channing Division of Network Medicine, Brigham and Women’s Hospital, Harvard Medical School, Boston, MA 02115, USA; 3 Boston Children’s Hospital, Boston, MA 02115, USA; 4 Division of Pediatric Pulmonary Medicine, UPMC Children’s Hospital of Pittsburgh, University of Pittsburgh, Pittsburgh, PA 15260, USA; 5 Rady Children’s Hospital, San Diego, CA 92123, USA

**Keywords:** asthma, ANCOVA, endophenotypes, genetics, pharmacology

## Abstract

**Objective:** Asthma is a complex and heterogeneous syndrome, making it hard to predict disease progression and suitable treatments. One strategy for reducing this uncertainty is to define genetic subtypes, or endophenotypes, that capture shared biological mechanisms. Most genome-wide studies, however, compare one subgroup against all others within a single cohort and rarely replicate their findings. We aimed to determine whether simultaneously modeling all asthma endophenotypes improves the discovery and replication of genetic associations compared with the standard one-versus-rest approach. **Methods:** We analyzed common single-nucleotide polymorphisms (SNPs) in the Childhood Asthma Management Program (CAMP) using an analysis of covariance (ANCOVA) across all severity-related endophenotypes, adjusting for age, sex, and ancestry principal components. SNPs showing genome-wide significance were tested for replication in the Genetics of Asthma in Costa Rican Children Study (GACRS). For comparison, we performed traditional one-versus-rest logistic regression analyses within each cohort, using identical covariates and endophenotype labels. **Results:** The ANCOVA identified 244 genome-wide significant SNPs in CAMP, of which six unique loci replicated in GACRS. In contrast, logistic regression recovered only four significant contrasts from those six loci in CAMP and replicated just one in GACRS. **Conclusions:** Our findings highlight genetic variants that are associated with asthma severity endophenotypes and demonstrate that modeling all clinical subtypes simultaneously can reveal biologically meaningful signals that are missed by standard pairwise design.

## 1. Introduction

Asthma is a complex condition that affects about 30 million Americans and about 300 million people worldwide [1]. Asthma is a multifactorial syndrome arising from diverse combinations of genetic variations and environmental exposures and comprising unique molecular mechanisms that result in marked disease heterogeneity [2,3,4]. Such heterogeneity is evidenced by substantial differences in disease triggers, progression, and treatment responses among affected individuals [5,6], underscoring the need to define and understand underlying endotypic differences. Conventional genome-wide association studies (GWAS) usually include all asthma patients in a single case group [7], implicitly assuming a shared genetic architecture that likely misses loci that contribute to specific subtypes of asthma. Recognizing these clinically meaningful subgroups has prompted efforts to incorporate endotypic information into genetic and genomic studies [8,9,10], highlighting the need for stratified or subtype-aware analytical methods.

Several efforts have been made to classify asthma patients into distinct severity subtypes to uncover the underlying biological mechanisms for severity [11,12,13]. However, GWAS of asthma severity endotypes remains largely unexplored, and existing studies usually reduce endotypes back into a case-control framework rather than treating them as true categorical outcomes. Since endotypes represent discrete categories, not a continuous trait, linear or binary comparisons are limited, particularly if more than one group shares the same genetic influences. The existing GWAS of endotypes have then taken a one-versus-one, case-control approach by testing each endotype separately against all other subjects with a simple logistic regression model [14,15,16,17,18,19]. This pairwise strategy multiplies the number of hypotheses, thus inflating the multiple-testing burden, making it harder to discover subtype-specific loci. Multivariate or multi-level association models have been increasingly recognized as more powerful alternatives to pairwise approaches [20,21,22]. Analytical approaches such as analysis of variance (ANOVA) and its covariate-adjusted form, analysis of covariance (ANCOVA), which can model all endophenotypes simultaneously and better capture shared or overlapping genetic influences, have yet to be fully explored in this context.

In this study, we investigate genetic variants that drive biological differences between clinically defined asthma endophenotypes. The endophenotypes used in this analysis were previously defined using principal component analysis (PCA) of baseline clinical features across three independent pediatric asthma cohorts (CAMP, PACT, and GACRS) [23]. Subjects were grouped into five ordinal categories (Q1–Q5) based on quintiles of their PC1 scores, which captured major variation in asthma severity and atopy. Across cohorts, PC1 loadings were dominated by markers of atopic status (IgE, positive skin test, etc.), lung function (FEV1/FVC ratio, peak expiratory flow, etc.), and related demographic factors (age, sex, age of onset, etc.), together explaining the majority of variance in baseline clinical presentation. Q1 represented children with milder disease and higher lung function, whereas Q5 included those with greater atopic burden, lower lung function, and other markers of more severe asthma.

This PCA-based framework provided a reproducible, quantitative definition of asthma endophenotypes that remained consistent across independent pediatric cohorts and was shown to predict corticosteroid treatment response. Similar dimension-reduction and clustering frameworks have previously been used to delineate asthma phenotypes and inflammatory endotypes, demonstrating that latent clinical structure can reveal biologically meaningful subgroups [24,25]. To identify which clinical endophenotypes are enriched or depleted for the risk allele, we apply ANCOVA. In genetic analysis, ANCOVA evaluates whether allele frequencies differ across multiple endophenotypes while adjusting for covariates such as ancestry or clinical factors. Unlike traditional one-versus-rest or pairwise logistic models, which compare each contrast separately, ANCOVA tests all groups simultaneously. This framework allows us to capture multi-cluster heterogeneity and directly evaluate subtype-specific differences in a statistically efficient way. ANCOVA not only reduces within-group error variance and increases statistical power, but also allows adjustment for key clinical and demographic covariates that could confound genetic associations. By avoiding the inflation inherent to multiple pairwise tests and instead evaluating all endophenotypes in a unified framework, ANCOVA facilitates the identification of both subtype-specific and shared loci, providing a biologically coherent understanding of asthma heterogeneity.

## 2. Methods

### 2.1. Study Populations

The analysis was first conducted in the Childhood Asthma Management Program (CAMP, discovery cohort) and then replicated in the Genetics of Asthma in Costa Rica Study (GACRS, replication cohort). CAMP [26,27], was a multicenter, randomized clinical trial of inhaled corticosteroids to prevent severe asthma exacerbations in 1041 children aged 5 to 12 years, with mild to moderate persistent asthma. GACRS [28] was an observational cross-sectional study of 1165 Costa Rican children aged 6 to 14 years with physician-diagnosed asthma and at least two respiratory symptoms or a history of asthma attacks in the previous year. Written parental consent and/or the subject’s assent were obtained for each study protocol and ancillary genetic testing. Study protocols were approved by local Institutional Review Boards at each recruitment site for both studies, and by the Institutional Review Board of Brigham and Women’s Hospital.

### 2.2. Genotyping and Quality Control

SNPs were assayed on high-density Illumina arrays (Illumina Inc., San Diego, CA, USA). CAMP subjects were genotyped on 550 K v3 and 610 Quad BeadChips while GACRS participants were genotyped on OmniExpress and Omni2.5 BeadChips. Array-specific quality control (QC) was conducted in PLINK v1.9 [29]. Samples were excluded for call-rate < 95%, absolute heterozygosity deviations > 0.20 from the cohort mean, sex discrepancies, Mendel error rates in pedigrees, or excess relatedness identified by pairwise identity-by-state sharing. Variants were removed if monomorphic, exhibited a call-rate that was <95%, had a minor-allele frequency (MAF) < 5%, or violated Hardy Weinberg equilibrium ($p<1\times 10^{-6}$). Cleaned datasets from each chip were merged using PLINK v1.9 and phased and imputed on the Michigan Imputation Server to the Haplotype Reference Consortium (HRC) panel [30]. Post-imputation QC was performed in PLINK v2.0 [29]. We excluded variants with a call rate < 95% or had a minor-allele frequency (MAF) < 5%, and removed samples with genotype missingness > 5%. Our analysis included 792 subjects from CAMP and 1030 subjects from GACRS after QC with 3384590 SNPs common between CAMP and GACRS.

### 2.3. Statistical Analysis

Five endophenotypes of asthma were defined using multivariate clinical characteristics [23]. Briefly, principal component analysis (PCA) was applied to baseline clinical features, and subjects were grouped into five ordinal categories (Q1–Q5) according to quintiles of their first principal component (PC1) scores. In prior work, this PC1 axis captured major variation in asthma severity and atopy and demonstrated reproducible endotype structure across three pediatric cohorts, including CAMP and GACRS [23]. For reproducibility, PCA loadings from CAMP are provided in Supplementary Table S1. Researchers may standardize their clinical variables and multiply them by this loading matrix to reproduce PC scores and assign endophenotypes. We treat the five endophenotypes as the five levels of a single categorical factor and test them simultaneously within one model. This framework accounts for between-cluster distinctions that are not apparent in binary splits and maintains the latent continuum captured by the PC1-derived endophenotype bins, while also reducing the need for multiple pairwise tests and the associated multiple-testing burden. We tested whether allele dosages differed across endophenotype groups using an ANCOVA model:

$$\mathrm{genotype}∼\mathrm{endophenotype}+\mathrm{age}+\mathrm{sex}+{\mathrm{PC}}_{1}–{\mathrm{PC}}_{10}$$

Covariates such as age, sex, and ancestry principal components (PC_1_–PC_10_) were included to account for stratification and avoid confounding effects from demographic factors. Model fit was summarized using the F statistics, which indicate the ratio of between-group variance to within-group variance. To identify which endophenotypes were driving overall associations, we conducted post-hoc pairwise group comparisons using Tukey’s Honestly Significant Difference (HSD) test. Genome-wide significance thresholds were applied to ANCOVA results, and Tukey’s contrasts were used to report endophenotype-specific allele frequency differences. The top replicated ANCOVA SNPs were then subjected to one-vs.-rest logistic regression:

$${\mathrm{endophenotype}}_{k}\;\left(1=k,\;0=\mathrm{others}\right)∼\mathrm{genotype}+\mathrm{age}+\mathrm{sex}+{\mathrm{PC}}_{1}–{\mathrm{PC}}_{10}$$

run separately for five endophenotypes. To further evaluate whether categorical ANCOVA signals reflected an underlying continuous severity axis, we applied ordinal regression using $PC_{1}$ severity scores to test for monotonic risk allele frequency trends across endotypes. All statistical analyses were conducted in R (version 4.4.2) using the aov(), lm(), and glm(). The F statistic, odds ratio, and *p*-values are reported.

### 2.4. Machine Learning Prediction

To evaluate predictive performance, we trained both Elastic Net and XGBoost classifiers using SNPs that passed a significance threshold of $p\leq 1\times 10^{-5}$ in CAMP (1976 variants), followed by LD clumping (windows of 250 SNPs with a step size of 50 variants, and retained variants with $r^{2}<0.5$) to yield 247 independent SNPs. Classifiers were trained in CAMP with cross-validation and assessed using one-vs.-rest receiver operating characteristic (ROC) across the five endophenotypes. For both models, hyperparameters were tuned using five-fold cross-validation within the CAMP cohort to optimize for AUC. The elastic net model was tuned for a range of regularization strengths (C) and L1 ratios, while the XGBoost model was tuned over key parameters including learning rate, tree depth, and regularization terms (L1/L2). Performance was then evaluated in the independent GACRS cohort.

## 3. Results

Table 1 summarizes the baseline characteristics of CAMP and GACRS asthma cohorts, stratified by endophenotypes. While the mean age was significantly progressively higher along with the order of endophenotypes in both cohorts, the FEV1 pre-bronchodilator percent predicted (preBDFEV1PP) was significantly progressively lower as the order of the endophenotypes increased, consistent with increased asthma severity. There was no significant difference in the participants’ sex across endophenotypes in either CAMP or GACRS. In CAMP, non-Hispanic white participants were more likely to be in endophenotype 1, while non-Hispanic Black participants were more likely to be classified in endophenotype 5.

**Figure 1:**
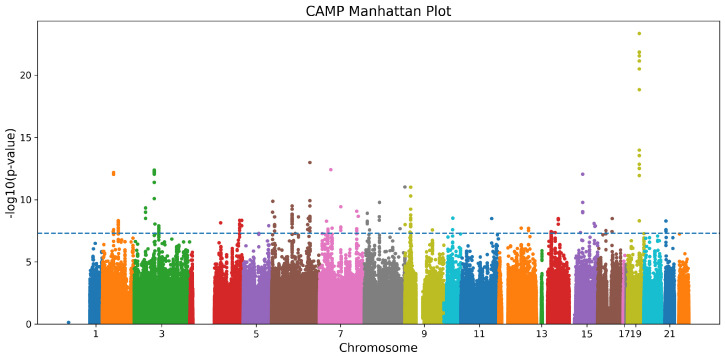
Manhattan plot for the CAMP cohort. Each point represents a SNP, with colors alternating by chromosome, and the dashed horizontal line indicates the genome-wide significance threshold.

The multivariable ANCOVA analysis was adjusted for age, sex, and the top ten genetic ancestry PCs in the CAMP cohort. Figure 1 shows the Manhattan plot from the ANCOVA models, and Table 2 summarizes the six LD-independent SNPs that reached genome-wide significance in CAMP and their replication statistics in GACRS. In the discovery cohort, CAMP, 244 SNPs were found to meet the genome-wide significance threshold of $5\times 10^{-8}$. After LD clumping, six unique SNPs remained significant (rs10964536, rs28892326, rs2823880, rs10086065, rs12448208, rs2754324) with ANCOVA F values 10.3–12.0 ($p\leq 5\times 10^{-8}$). The tables also show the F score measuring overall heterogeneity, the odds ratio, and post hoc comparisons revealing significant group differences. Applying the identical ANCOVA model to GACRS confirmed a nominal association (p<0.05) for all six loci.

In CAMP (Table 2), the top signal was rs10964536, located on chromosome 9 (F value = 12.03). Endophenotype-specific contrasts indicated that allele dosage differed significantly in endophenotypes 4 vs. 1, 5 vs. 1, and 4 vs. 3. This indicates that carriers are enriched in the higher-order endophenotypes. In CAMP and GACRS, these high-order endophenotypes correspond to PC1 quintiles Q4–Q5, which were characterized by lower baseline lung function, higher IgE and eosinophil counts, and greater atopic burden compared with Q1–Q2 [23]. The same marker replicated in GACRS with F = 3.23 (*p* = 0.0121) and a significant 5 vs. 1 contrast. For rs28892326, rs2823880, and rs10086065, ANCOVA in CAMP yielded F values 11.9–11.4 with significant 4 vs. 1 and 5 vs. 1 comparisons that survived Tukey correction (*p* ≤0.05). Each locus replicated nominally in GACRS (p<0.05); rs2823880 showed the strongest replication (F = 5.12, $p=4\times 10^{-4}$) with multiple post-hoc contrasts (4 vs. 1, 4 vs. 2, 4 vs. 3). rs12448208 also separated 2 vs. 1 and 4 vs. 1 in CAMP and displayed nominal replication in GACRS. Similarly, rs2754324 distinguished endophenotype 4 from 1 and 3 in CAMP, with nominal replication for 5 vs. 3 in GACRS.

Across the six top SNPs (five endophenotypes = 30 possible one-vs.-rest tests), logistic regression (Table 3 showed that 12 out of 30 contrasts (40%) reached a significance of 0.05 before multiple-test correction. After Bonferroni adjustment (alpha = 0.05/30 = 0.0016), only 4 (16%) contrasts remain significant. For logistic regression after Bonferroni correction in GACRS for 30 contrasts (α∼0.0016), only rs2823880 remained statistically significant for endophenotype 4 vs. rest. These results underscore that conventional one-vs.-rest logistic regression captures only a fraction of the associations detected by ANCOVA, which tests all endophenotypes simultaneously and therefore retains greater power to identify multi-group differences.

**Figure 2:**
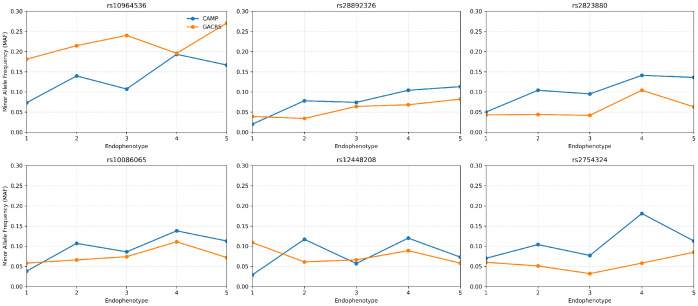
MAF patterns across endophenotypes for CAMP and GACRS.

The minor-allele frequencies differed substantially across the endophenotypes and reflected the post-hoc contrasts identified by ANCOVA (Figure 2). For all six loci in CAMP, the minor-allele dosage was significantly higher in high-severity endophenotypes. Notably, rs10964536 shows a strong enrichment in endophenotypes 4 and 5 compared with 1 as well as for 4 vs. 3, with delta MAF upto 12%. Similarly, rs28892326 (delta MAF∼8–9%), rs2823880 (delta MAF∼9%), and rs10086065 (delta MAF 7.5–10%) also show enrichment for endophenotype 4 and 5 vs. 1. rs12448208 (delta MAF∼9%) shows an early severity enrichment for detecting endophenotypes 2 and 4 compared with 1. rs2754324 (delta MAF 10–11%) showed strongest variation in endophenotypes 4 compared with 1 and 3. In GACRS, five of the six loci showed the same directional trend toward higher minor-allele frequency in more severe endophenotypes, with rs12448208 as the only exception, which did not replicate and showed an opposite pattern.

**Figure 3:**
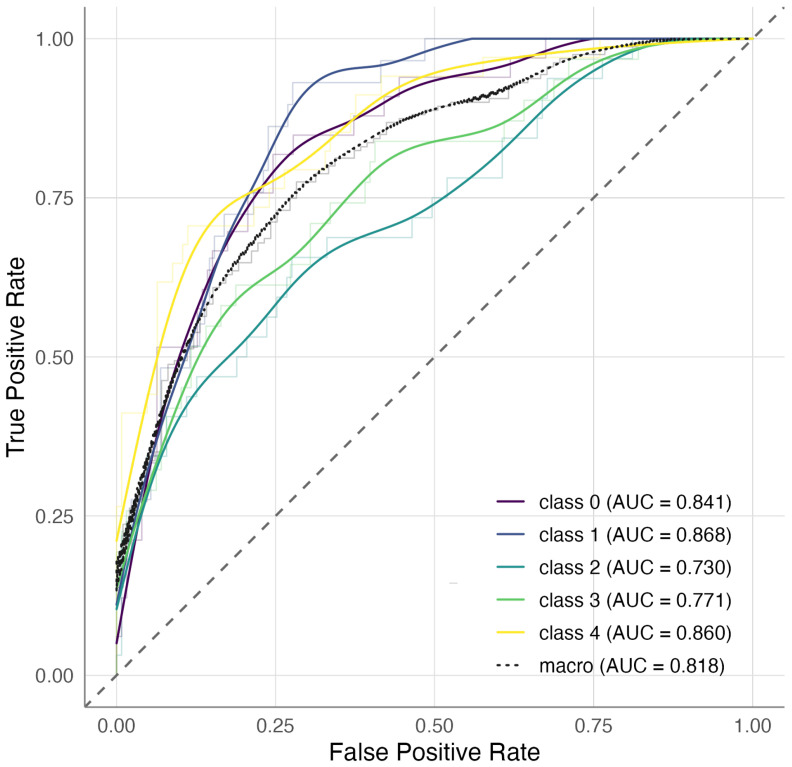
One-versus-rest ROC curves for endophenotype prediction in CAMP using Elastic Net. The average AUC was 0.82, with class-specific AUCs ranging from 0.73 to 0.87.

To further verify that categorical ANCOVA signals follow a continuous severity axis, we tested the six genome-wide discovery loci with an ordinal trend model using PC1 scores. We noticed an increase in each SNP’s risk allele in the same direction as asthma severity in both cohorts, with modest *p*-values (range of 0.001–0.1), supporting a severity trend and the increased power from discrete endophenotype grouping. In CAMP, the Elastic Net classifier achieved good discrimination across severity endophenotypes with per-class AUCs ranging from 0.73 to 0.87 and an average AUC of 0.81 (Figure 3). XGBoost performed comparably for some classes but was less stable overall (AUCs ≈0.56–0.82) In contrast, external evaluation in GACRS showed no generalization, with both Elastic Net and XGBoost models performing at chance level across all classes (AUCs ≈0.48–0.53).

## 4. Discussion

In this study, we evaluated the genetics of asthma severity endophenotypes using a novel ANCOVA approach. Specifically, we employed ANCOVA to test whether allele frequencies differ across clinically defined severity endophenotypes and contrasted this with a more conventional one-versus-rest logistic regression framework. Applying these methods in two independent pediatric asthma cohorts (CAMP and GACRS), we found that ANCOVA detected 244 genome-wide significant SNPs in CAMP, with six LD-independent loci, all of which replicated in GACRS. By comparison, the one-versus-rest logistic models identified fewer significant contrasts, highlighting the improved sensitivity and power of ANCOVA for capturing genetic determinants across multiple severity endophenotypes simultaneously. Further LD-clumping resulted in six unique loci that confirmed cross-study screening. These six loci were then subjected to five logistic regressions using a one-vs.-rest approach, which revealed only four significant contrasts, while post-hoc group differences from ANCOVA revealed 13 significant contrasts in CAMP. In GACRS, eight contrasts were identified by ANCOVA and only one survived multiple-testing correction. This confirms that ANCOVA outperforms multiple one-vs.-rest logistic tests in power and cross-cohort consistency due to fewer tests, contrasts encoded within one model, and better use of within-cohort heterogeneity. Every ANCOVA significant contrast resulted in ≥7.5% delta MAF in CAMP and ≥4% delta MAF in GACRS. Moreover, five out of six loci showed the same upward MAF gradient toward severe endophenotypes in both cohorts, except for SNP rs12448208. Risk allele frequencies increase with severe asthma endophenotypes, and MAF patterns reinforce the genetic findings across endophenotypes.

The clinically defined severity endophenotype was used to identify allele-frequency differences using ANCOVA with Tukey post-hoc group contrasts. The F score captures any heterogeneity across endophenotypes, and Tukey contrasts then identify driving endophenotypes. One-vs.-rest logistic regressions do not reveal group contrasts and suffer from increased burden due to multiple-testing correction. ANCOVA with categorical endophenotypes increases the discovery power and reveals the varying contrasts. Noteably, an attempt to address dimensionality and power [31] uses logit-transformed allele frequencies and models their interaction to explain cluster differences via simulations. While this method offers advantages in scalability, it lacks statistical inference and biological interpretability. By contrast, our approach employs ANCOVA to explicitly test varying allele frequencies across endophenotypes, producing biologically interpretable inferences and enabling cross-cohort validation. This suggests that ANCOVA can detect allele-frequency differences across groups and, by reducing the number of tests, may mitigate some of the sample size limitations inherent to endophenotype analyses.

Across the six loci, five map within or near transcribed genes or open reading frames, while one SNP (rs10964536) lies in an intergenic region with no clear functional annotation. Among these, rs28892326 is located within *DGKI*, which regulates airway smooth muscle proliferation and remodeling [32]. The remaining loci fall within genes or non-coding regions with limited or indirect links to airway or immune biology and therefore require further study to clarify their relevance. The variant rs2823880 is located within *MIR99AHG*, the host gene of the *miR-99a/let-7c/miR-125b-2* cluster [33,34]. The variant, rs12448208, is located near *SNX20* [35]. Overall, these patterns suggest that a subset of loci has plausible biological relevance but requires additional validation to determine their role in asthma.

Beyond association testing, we explored whether the top SNPs could stratify clinical endophenotypes using machine-learning classifiers. In CAMP, Elastic Net models trained on 247 LD-pruned SNPs achieved good within-cohort discrimination (average AUC = 0.81), while XGBoost performed less consistently (Figure 3). However, when applied to the external GACRS cohort, neither method was generalized, with AUCs close to 0.5 in all endophenotypes. Notably, using preselected SNPs based on marginal association ($p\leq 1\times 10^{-5}$) may contribute to model overfitting, as feature selection was informed by the same dataset used for model training. Although cross-validation was applied, this practice can inflate apparent predictive performance within the discovery cohort and limit generalizability across independent samples.

Our study had limited statistical power due to the small sample size of the endophenotype groupings, and larger, diverse asthma cohorts will therefore be required both to replicate these associations and to determine the true clinical relevance of the implicated loci across populations. Further, severity endophenotypes were derived using baseline clinical features, and longitudinal reassignment may change genotype-severity endophenotype mapping. Our results indicate that risk alleles are more common in severe asthma endophenotypes, and a severity-weighted polygenic risk score incorporating these endophenotype-specific variants may enhance the prediction of exacerbation risk and corticosteroid response. Additionally, SNP feature selection was performed on the full CAMP dataset prior to model training, which introduces the possibility of information leakage and may inflate within-cohort cross-validated performance. Although we attempted external validation in GACRS, the lack of model generalization highlights the limited transferability of CAMP-derived predictors and underscores the need for larger cohorts and nested feature-selection frameworks in future work.

In summary, our study indicates that ANCOVA applied to clinically defined asthma severity endophenotypes provides a complementary approach to logistic regression, enabling detection of group-level allele-frequency differences in settings where sample size is limited. By testing allele-frequency differences across categorical endophenotypes, ANCOVA identified genome-wide associations that replicated across cohorts and showed gradients consistent with asthma severity. Several loci were located in genes and regulatory regions related to inflammation, airway remodeling, and immune function. Elastic Net captured within-cohort variation but did not generalize across cohorts, underscoring limitations in transferability. Larger and more diverse studies, together with functional analyses, will be needed to confirm these findings and clarify their potential relevance for risk prediction and treatment.

## Figures and Tables

**Table 1:** Demographic characteristics by endophenotype.

	CAMP Endophenotypes						GACRS Endophenotypes					
Characteristics	1	2	3	4	5	*p*-Value	1	2	3	4	5	*p*-Value
N	171	154	168	163	177	–	207	205	204	207	207	–
Age (mean)	6.96	7.87	9.04	9.87	10.88	<2 × 10^−16^	8.25	8.66	9.15	9.82	9.95	<2 × 10^−16^
Sex (% F)	47%	38%	39%	37%	34%	0.195	45%	42%	40%	36%	43%	0.38
PreBD FEV1PP	105.68	96.86	94.11	90.04	80.11	<2 × 10^−16^	115.24	105.56	100.59	93.44	81.13	<2 × 10^−16^
Race/ethnicity (%)						0.0008	100% Hispanic					–
White	84	72	74	68	63							
Black	6	14	11	16	11							
Hispanic	5	6	7	8	16							
Other	5	8	8	8	10							

**Table 2:** ANCOVA results for top SNP associations in CAMP and GACRS cohorts.

SNP	Region (GRCh38)	CAMP			GACRS		
		F	*p*-Value	Post-Hoc ^†^	F	*p*-Value	Post-Hoc ^†^
rs10964536	9p21.3	12.03	$1.71\times 10^{-9}$	4–1, 5–1, 4–3	3.23	0.0121	5–1
rs28892326	7q33	11.92	$2.11\times 10^{-9}$	4–1, 5–1	3.04	0.0166	5–2
rs2823880	21q21.1	11.43	$5.09\times 10^{-9}$	4–1, 5–1	5.12	0.0004	4–1, 4–2, 4–3
rs10086065	8p23.1	11.42	$5.23\times 10^{-9}$	4–1, 5–1	2.53	0.0390	4–1
rs12448208	16q12.1	10.35	$3.59\times 10^{-8}$	2–1, 4–1	2.81	0.0246	5–1
rs2754324	9p22.2	10.29	$4.00\times 10^{-8}$	4–1, 4–3	2.73	0.0282	5–3

^†^ Tukey-corrected pairwise contrasts that remained significant within each cohort.

**Table 3:** One-vs.-rest logistic regression for each asthma endophenotype in CAMP and GACRS cohorts, showing odds ratios (OR) and *p*-values for the risk allele within each endophenotype compared to all others.

SNP	Endophenotype vs. Rest									
	1		2		3		4		5	
	CAMP	GACRS	CAMP	GACRS	CAMP	GACRS	CAMP	GACRS	CAMP	GACRS
rs10964536	1.97 (0.0010)	1.37 (0.749)	0.97 (0.841)	1.04 (0.273)	1.30 (0.129)	0.86 (0.029)	0.65 (0.0028)	1.21 (0.168)	0.78 (0.088)	0.70 (0.0053)
rs28892326	3.44 (0.0007)	1.61 (0.0304)	1.00 (0.995)	1.86 (0.541)	1.05 (0.815)	0.87 (0.077)	0.75 (0.098)	0.81 (0.323)	0.68 (0.021)	0.62 (0.019)
rs2823880	1.95 (0.0031)	1.47 (0.149)	1.01 (0.952)	1.45 (0.100)	1.10 (0.590)	1.55 (0.136)	0.75 (0.054)	0.44 ($4\times 10^{-5}$)	0.77 (0.085)	0.93 (0.733)
rs10086065	2.24 (0.0016)	1.44 (0.374)	0.90 (0.550)	1.22 (0.818)	1.10 (0.581)	1.05 (0.117)	0.70 (0.021)	0.57 (0.0029)	0.86 (0.321)	1.07 (0.746)
rs12448208	2.56 (0.0022)	0.60 (0.183)	0.66 (0.017)	1.35 (0.373)	1.37 (0.160)	1.22 (0.0068)	0.63 (0.0083)	0.81 (0.279)	1.06 (0.755)	1.44 (0.111)
rs2754324	1.56 (0.0288)	0.93 (0.558)	1.05 (0.798)	1.16 (0.0162)	1.41 (0.077)	2.05 (0.762)	0.56 (0.0001)	0.98 (0.946)	0.96 (0.798)	0.58 (0.0088)

## Data Availability

The raw data supporting the conclusions of this article will be made available by the authors on request.

## Supplementary Materials

The following supporting information can be downloaded at: https://www.mdpi.com/article/10.3390/jpm16010032/s1, Table S1: Principal component loadings for PC1–PC3 in CAMP; Figure S1: PCA-based visualization of asthma severity endophenotypes (a) CAMP cohort; (b) GACRS cohort.
