# Supplementary material for: A Categorical ANCOVA Approach to Severity Endophenotype-Specific Genome-Wide Association Studies in Childhood Asthma
Source: J Pers Med. 2026 Jan 5;16(1):32. doi: 10.3390/jpm16010032 (PMC12842872; doi:10.3390/jpm16010032)
Supplement: Supplementary file 1 [file jpm-16-00032-s001.zip › jpm-3959839-supplementary.pdf]

Supplementary Table S1. Principal component loadings for PC1–PC3 in CAMP.

Category	Variable	PC1	PC2	PC3
**DEMOGRAPHIC**	SEX	–0.051651	–0.157550	0.13506
	RACE	0.10567	–0.022500	0.084805
	AGE	0.38134	–0.355960	0.040452
	AGE at onset	0.13418	–0.296570	0.11224
**SMOKE EXPOSURE**	IUS	–0.024765	0.07651	–0.123030
	ETS	–0.012443	0.032474	–0.106400
**OTHER**	Blood lymphocyte %	–0.071895	0.17488	0.618
	Blood neutrophil %	–0.021590	–0.207450	–0.639590
**ATOPY**	Atopic dermatitis	–0.013766	–0.009297	0.010224
	Any positive skin test	0.27692	–0.055452	0.07101
	Hay fever	0.12704	–0.121540	0.043688
	IgE (log10)	0.35232	–0.017108	0.095293
**EOSINOPHILS**	Blood eosinophils (log10)	0.2724	0.086852	0.14706
**LUNG FUNCTION**	Bronchodilator response	0.32434	0.37723	–0.121190
	FEV1/FVC ratio pre-bronchodilator	–0.371650	–0.294000	0.15858
	Peak Expiratory Flow pre-bronchodilator	0.22234	–0.444310	0.081623
	FEV1% predicted pre-bronchodilator	–0.376020	–0.294670	0.082378
	FVC % predicted post-bronchodilator	–0.121640	0.030419	–0.068433
**BMI**	Waist–hip ratio	–0.071443	0.12224	–0.166400
	BMI	0.19263	–0.273840	–0.107810
**SYMPTOMS**	SABA use last 6 months	0.16131	–0.022939	0.068629
	Nocturnal symptoms	0.000372	0.062676	–0.000258
**EXACERBATION**	Hospitalized	0.085932	0.19219	–0.041899
	ER visit	0.020854	0.092113	–0.036881

We provide the loading vectors for PC1–PC3 derived from the randomized clinical trial cohort, CAMP, which serves as the reference dataset for endotype derivation due to standardized baseline phenotyping and minimal confounding. These loadings allow external investigators to project their own standardized clinical variables into the same PCA space for endophenotype assignment.

(a) CAMP cohort

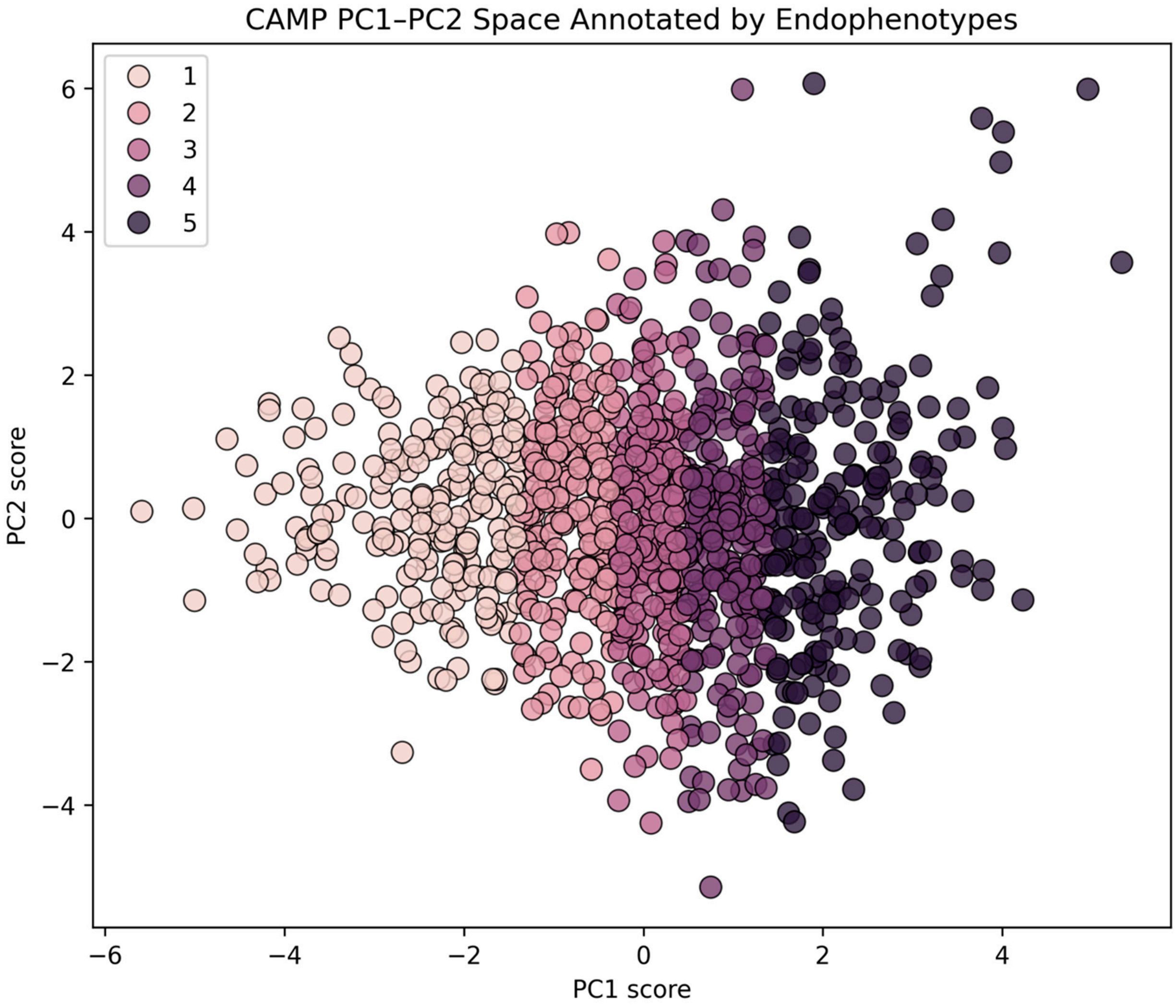

(b) GACRS cohort

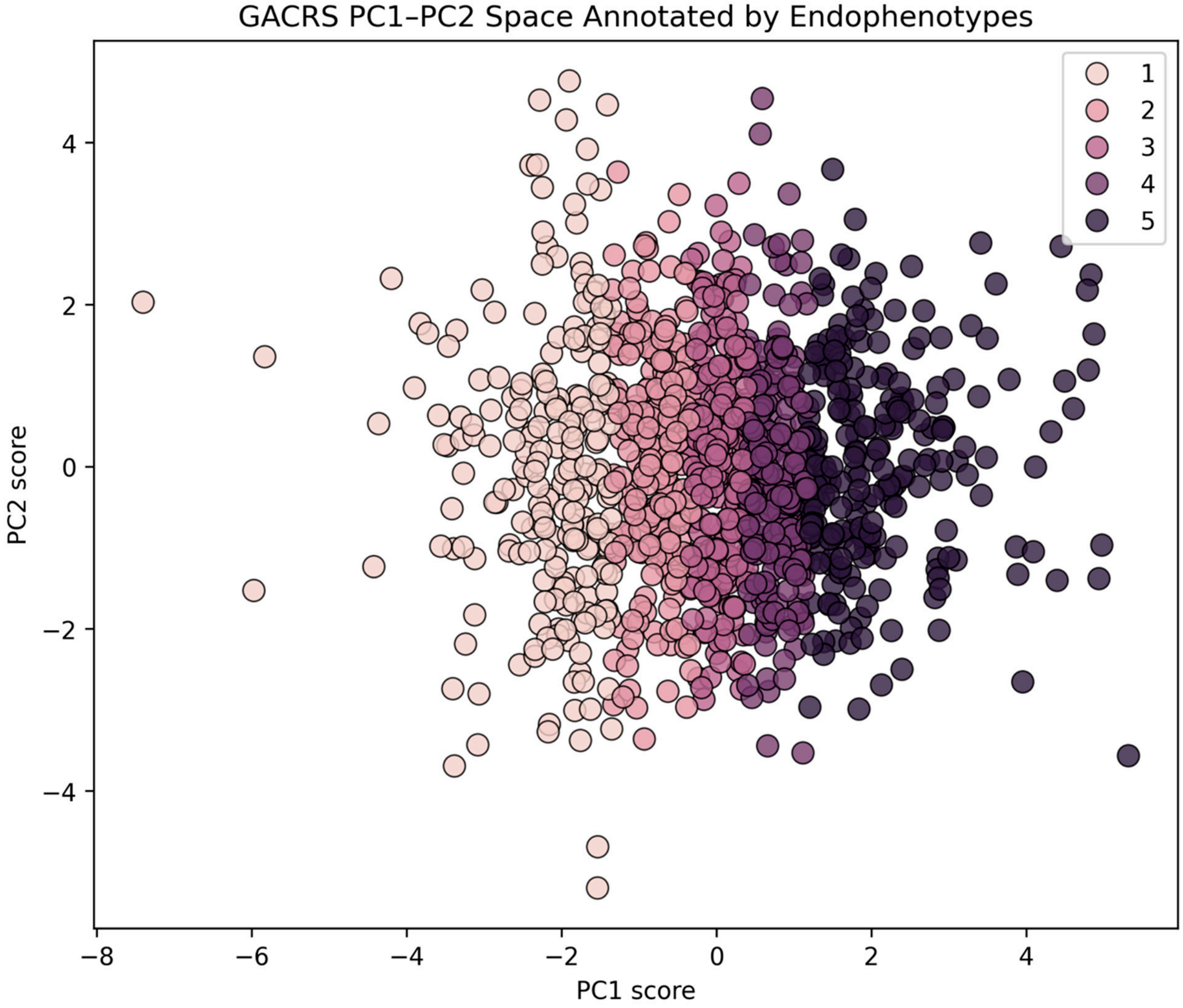

Supplementary Figure S1. PCA-based visualization of asthma severity endophenotypes. Principal component analysis (PCA) of subjects in the (a) CAMP cohort and (b) GACRS cohort, plotted using PC1 and PC2. Points represent individual subjects, colored by endophenotype assignment. The separation along PC1 reflects the dominant variance driven by input clinical features.
